# The effect of full length and mature NAG-1 protein overexpression on cytotoxicity of celecoxib, tamoxifen and doxorubicin in HT1080

**Published:** 2010

**Authors:** S Barezi, N. Fahham, M. Seyedabadi, S.N. Ostad, M.H. Ghahremani

**Affiliations:** 1 Department of Pharmacology-Toxicology, Faculty of Pharmacy, Tehran University of Medical Sciences; 2 Department of Biotechnology, Pasture Institute of Iran; 3 Department of Pharmacology, School of Medicine; 4 Department of Molecular Medicine, School of Advanced Medical Technologies, Tehran University of Medical Sciences, Tehran, Iran

**Keywords:** Cytotoxicity, Drug resistance, NAG-1, Cancer chemotherapy

## Abstract

**Background and the purpose of the study:**

Non-steroidal anti-inflammatory drug-activated gene-1 (NAG-1) is involved in inflammation, apoptosis/survival and tumorigenesis as well as resistance to chemotherapy. NAG-1 protein is synthesized as pro-peptide, cleaved and secreted as mature protein. Regulation of NAG-1 is not completely discovered and increased level of NAG-1 has been reported in many cancers. The expression of NAG-1 in cancer cells could affect the progression of tumor growth. In addition the secretion of full length and mature forms of NAG-1 can influence cell proliferation in other cells. In this study the role of full length and mature forms of NAG-1 on viability of HT-1080 and MCF-7 cells were evaluated, and the cytotoxicity of celecoxib, indomethacin, tamoxifen and doxorubicin in HT1080 cells stably expressing NAG-1 were also tested.

**Methods:**

Full length and mature NAG-1 was cloned from cDNA library of HCT116 cells and stably transfected in HT1080 cells. Cells were treated with different concentrations of indomethacin, celecoxib, tamoxifen and doxorubicin and viability was assessed by MTT assay. The effect of conditioned medium of NAG-1 expressing cells on proliferation of MCF-7 and HT1080 cells were also tested.

**Results:**

The growth curves of HT1080 cells expressing full length and mature NAG-1 were not different. The viability of HT1080 cells expressing NAG-1 in the presence of indomethacin, doxorubicin and tamoxifen compared to untransfected cells was higher. The proliferation of HT1080 and MCF-7 cells were inhibited by conditioned medium of NAG-1 expressing cells in 24 and 48 hrs.

**Major conclusion:**

NAG-1 expression enhances drug resistance to tamoxifen, indomethacin and doxorubicin in HT1080. In addition, condition medium of NAG-1 expression cells inhibits proliferation in MCF-7 and HT1080 cells. Thus, NAG-1 expression can induce drug resistance in NAG-1 expressing cells and inhibition of viability in non expressing cells. Thus, NAG-1 is suggested as a marker for effective cancer chemotherapy and tumor progression.

## INTRODUCTION

Non-steroidal anti-inflammatory drug-activated gene-1 (NAG-1), (also known as MIC-1, GDF-15, PTGFβ and PLAB) is a distinct member of TGFβ super family which is involved in different cellular processes like inflammation, apoptosis/survival and tumorigenesis as well as resistance to chemotherapy and radiotherapy (1, 2). NAG-1 protein is synthesized as a 308-amino acid pro-peptide. The mature form is secreted as a 30-kDa dimeric protein after furin like cleavage at the RXXR site and formation of many disulfide bonds between cysteine residues in the endoplasmic reticulum (2). It has been shown that NAG-1 expression increased during inflammation, tissue injury and malignancy. Increased NAG-1 expression is a common feature of many cancers, including breast, colon, pancreas and prostate cancers, suggesting NAG-1 as a tumor biomarker. Regulation and expression of NAG-1 is not completely discovered but it is known that NAG-1 is one of target genes of p53 activating pathways and its expression is induced either through p53 and/or EGR-1 related pathways (3–6).

The function and signaling pathway of NAG-1 in cancer progression and treatment still remain unclear. Although there are in vitro studies suggesting anti-proliferative and pro-apoptotic functions of NAG-1 in cancers of colon, breast and prostate (7), a number of studies have shown that NAG-1 is involved in progression of breast, gastric, prostate and colorectal carcinomas (8–11). Besides, NAG-1 serum concentration is elevated in metastatic cancer. In this regard, it has been shown that NAG-1 induces invasiveness of human gastric cancer cells via multiple pathways including ERK1/2 pathway, urokinase plasminogen activator upregulation and MMP pathway (12, 13). NAG-1 over-expression may confer resistance of cancer cells to chemotherapy and radiotherapy in different cell types (1, 2).

Therefore, the expression of NAG-1 in cancer cells could affect the progression of tumor growth. In addition, the secretion of full length and mature forms of NAG-1 can influence cells in distance tissue and modulate their proliferation. Thus, in this study the role of full length and mature forms of NAG-1 on viability of HT-1080 and MCF-7 cells were evaluated. Furthermore, the change in cytotoxicity of celecoxib, indomethacin, tamoxifen and doxorubicin in HT-1080 cells stably expressing NAG-1 was tested.

## MATERIAL AND METHOD

### 

#### Cell lines

Human cancer cell lines HT1080 (human fibrosarcoma), MCF7 (human breast carcinoma), HCT116 (human colorectal carcinoma) were purchased from the National Cell Bank (Pasture institute of Iran, Tehran). Cells were cultured in RPMI 1640 medium (Biosera, England) supplemented with 10% heat-inactivated fetal bovine serum (FBS; Biosera, England) and antibiotics (100 U/ml penicillin and 100 µg/ml streptomycin; Gibco, USA) and incubated in a 37 °C,5% CO2 in humidified atmosphere.

#### Cloning of NAG-1 Full length and mature form

Total RNA was isolated from HCT116 cells using Tripure (Roche, Germany) and 2µg were used for cDNA synthesis using Revert Aid M-MuLV reverse transcriptase (Fermentas, Ukraine) and random hexamer (Fermentas, Ukraine). The cDNA for NAG-1 full-length coding region was amplified with primer pairs containing *Kpn*I or *EcoR*I restriction site (underlined); *Forward: 5'TCCGGTACCTGCACAGCCATG3’, Reverse: 5'GAAGGACCAGGAATTCTCATATGCAG3 ’.* The PCR product was digested with *EcoR*I and *Kpn*I (Fermentas, Ukraine) and then ligated into pCDNA3.1. The mature NAG-1 was generated using PCR and following, *Forward: 5'AGCATGCGAGCGCGCAACGGG3’, and Reverse: 5'GCACAGTGGAAGGTACCGGACTGCTCATA3’* containing the C-terminal 112 amino acids of full length protein. A start codon with Kozak sequence is engineered (underlined) for proper expression of protein. The PCR product was cloned into pCR2.1 TOPO (Invitorgen, USA) and subcloned into pCDNA3.1 in *Hind*III and *Xho*I sites. The generated plasmids were analyzed by agarose gel electrophoresis and the sequences were verified by DNA sequencing.

#### Stable transfection

HT1080 cells were plated at 500×103 cells/well in 6 well and cultured for 24 hrs. Cells were then transfected with full NAG-1pCDNA3.1 or mature NAG-1pCDNA3.1 or pCDNA3.1plasmids using FUGENE 6 (Roche, Germany) and cultured in RPMI. After 24 hrs, cells were plated1:2 and treated with Geneticin (500 µg/ml) for 2 weeks. Stable clones for full length and mature forms were collected as stable pool and used for further analysis. mRNA expression was analyzed using RT-PCR and specific primer pairs targeted to full length or mature form of NAG-1 constructs.

#### Drug resistance and cytotoxicity assay

HT1080 untransfected cells, full NAG-1 stable cells and mature NAG-1 stable cells were plated at 10×103 cells/well in 96 well (six replicate for each group, n=6) for 24 hrs. Then cells were treated by indomethacin (Hakim pharmaceutical co., Iran), celecoxib (synthesized in Medicinal Chemistry Lab, Faculty of Pharmacy, Tehran University of Medical Sciences, Iran), tamoxifen (Iran hormone, Iran) and doxorubicin (Ebeve, Austria) in different concentrations for 24 hrs and then MTT assay was performed.

To assess the effect of secreted full length and mature NAG-1 protein, HT1080 stable cell lines expression full length and mature protein were cultured for 24 hrs. Untransfected HT1080 and MCF7 cells were plated at 10×103 cells/well in 96 well for 24 hrs. Then upper medium of mature NAG-1 stable cell line and full NAG-1 stable cell lines were added to untransfected HT1080 and MCF7 cell lines. As control, cells without changing the medium were used. The MTT assay was performed after 24, 48, 72 hrs incubation.

#### Statistical analysis

The results were analyzed using one way ANOVA followed by Tukey-Kramer post test. The *P* value less than 0.05 (p<0.05) was considered significant.

## RESULTS

### 

#### Cell proliferation in stable lines

A comparison of the growth curves of HT1080 cells stably transfected with full length or mature NAG-1 with untransfected cells indicate no significant difference in their proliferation patterns (Figure 2), although the transfected cell lines were more resistance to acidic pH and contact inhibition.

**Figure 1:**
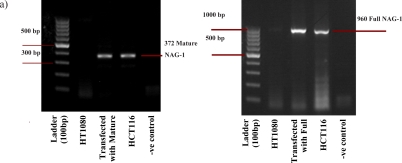
The mRNA transcripts of full length and mature form of NAG-1 were analyzed by RT-PCR in HT1080 and stable cell lines of HT1080. HCT116 cells were used as positive control.

**Figure 2:**
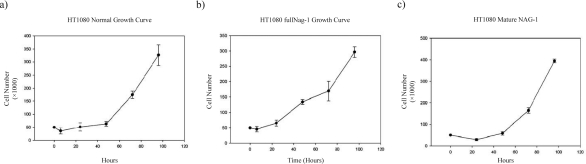
The growth curve of HT1080 cells expressing NAG-1 protein. (a–c) Stable lines and untransfected cells were seeded at 5×10^4^ in 24well plates and counted at different time using trypan blue dye exclusion. Data from three wells are presented as Mean ± SD (n=3).

#### Effect of NAG-1 expression on Drug resistance

**Figure 3:**
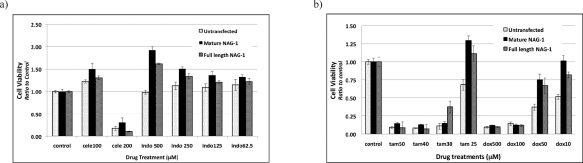
Effect of NAG-1 expression on drug cytotoxicity in HT1080 cells. HT1080 cells stably expressing full length and mature form NAG-1as well as untransfected HT1080 were seeded at 10×10^3^ cells/well in 96 well for 24 hrs. Cells were then treated with different concentration of (a) indomethacin (indo), celecoxib (cele), (b) tamoxifen (tam) and doxorubicin (dox) for 24 hrs and MTT assay was performed. Data from six replicate (n=6) are presented as Mean ± SD.

Indomethacin (62.5-500 µM) and Celecoxib (100 µM) showed no toxicity on HT1080 cells (Figure 3a). Interestingly the HT1080 cells expressing NAG-1 were more viable than untransfected cells. The viability was higher in cells expressing mature NAG-1 and at high doses of indomethacin (Figure 3a) Doxorubicin (10-500M) and tamoxifen (25–50M) inhibited HT1080 cell viability in a dose dependent manner (Figure 3b). When Mature and/ or full NAG-1 were expressed, HT1080 cells were more resistance to doxorubicine and tamoxifen at low doses (Figure 3b). Similar to COX inhibitors, the viability was higher when mature NAG-1 is expressed. High doses of doxorubicin and tamoxifen did not show any significant difference in transfected and untransfected cells.

#### Effect of full length and mature form on viability of HT1080 and MCF-7 cells

**Figure 4:**
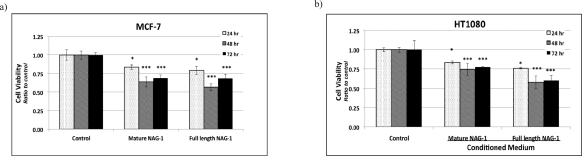
Effect of NAG-1 protein on viability of HT 1080 and MCF-7 cells. MCF-7 and HT1080 cells were seeded at 10 ×10^3^ cells/well in 96 well for 24 hrs. The conditioned medium of HT1080 cells stably expressing full length and mature form of NAG-1 were collected after 24 hrs and added to MCF-7 (a) and untransfected HT1080 (b) cells. The viability of cells incubated with conditioned medium was assessed in 24, 48 and 72 hrs by MTT assay. Data from six replicate (n = 6) are presented as Mean ± SD (* p < 0.05, *** p < 0.001, compared to control).

Since NAG-1 protein is secreted to the medium, the effect of NAG-1 protein on untransfected HT0180 and MCF-7 cells was investigated by incubating cells in conditioned medium of stable cell lines expressing mature and full length protein. Results indicate that conditioned mediums containing NAG-1 protein reduced both cell lines viability by 20% in 24 hrs (Figure 4). This effect was increased to 40-50% in 48 hrs for both cell line. The cytotoxicity effect was not different for mature and full length protein (Figure 4).

## DISCUSSION

In this study we have generated stable HT1080 cells expressing full length and mature forms of NAG-1 protein. Results indicate that expression of NAG-1 does not significantly alter growth curve of HT1080 cells compared to untransfected cells. On the other hand, addition of conditioned medium containing NAG-1 protein inhibited HT1080 and MCF-7 proliferation. This effect is in agreement with the findings which suggest a tumor suppressor role for NAG-1 mature form (2, 5, 6, 9, 11, 14). It has been reported that NAG-1 can inhibit proliferation and/or induce apoptosis in a p53 dependent manner (5, 6). HT1080 and MCF-7 cells are wild type for p53 (15, 16). Therefore, as it was found, in these cells NAG-1 can inhibit proliferation. However, there was not any significant slow proliferation in stable NAG-1 expressing cell, suggesting perhaps a compensation mechanism in these cells such as upregulation of MMP pathway. A comparison of full length and mature form indicate similar results for both forms. Considering the presence of MT1-MMP in HT1080 cells that can cleave pro-form of NAG-1 to mature form, the full pro-form can be processed to mature form and affect the cells.

It has been shown that NAG-1 expression can alter the response to chemotherapeutic agents (17, 18). Moreover, in colorectal HCT116 cancer cells silencing NAG-1 will sensitize tumor to oxaliplatin, 5-fluorouracil, and SN38 (18). We have evaluated the cytotoxicity of tamoxifen and doxorubicin in HT1080 cells expressing NAG-1 protein. Results showed that at low doses, NAG-1 expression, in full length or mature form, increases viability of cells treated with tamoxifen or doxorubicin (Figure 3), suggesting an increase in drug resistance which is blocked at high concentrations. Furthermore, there is a reverse correlation between COX-2 and NAG-1 expression in colorectal tumors^19^. When the effect of COX inhibitors on viability of HT1080 cells was investigated, indomathacin had no cytotoxicity even at high doses (500 µM) and celecoxib exerted growth inhibition in 200 µM (Figure 3). Interestingly, NAG-1 expressing HT1080 cells increased proliferation and viability in the presence of indomethacin. This effect is dose dependent and reaches to 2 fold elevation at 500µM in cells expressing mature form. In the presence of celecoxib, a slight increase in viability was observed in NAG-1 expressing cells. Thus, expression of NAG-1 make HT1080 cells susceptible to drug resistance. Although the resistance is not present at high doses of these drugs, results of this study indicate that NAG-1 expression increases risk of drug resistance. Furthermore, because of secretory nature of NAG-1, release of this protein can affect other cells in the body. It has been shown that in early stage of cancer, NAG-1 can induce tumor apoptosis, and at late stages, NAG-1 production will help metastases and tumor progression (9). Furthermore, elevated level of NAG-1 in patient with glioblastoma has been correlated with shorter survival (20). Therefore, NAG-1 expression could induce tumor progression and perhaps drug resistance in cancer (21).

## CONCLUSION

Taken together, an enhanced drug resistance to tamoxifen, indomethacin and doxorubicin in HT1080 cells expressing NAG-1 is reported. Furthermore, condition medium from NAG-1 expression cells inhibits cell proliferation in MCF-7 and HT1080 cells. Thus, understanding NAG-1 expression and function provides information for effective cancer chemotherapy.
